# Dr. Webster on the Statistics and Pathology of Insanity

**Published:** 1848-01-01

**Authors:** 


ART. VI.—(1.) *Statistics and Pathology of Mental Diseases.* By JOHN WEBSTER, M.D., F.R.S., &c.

(2.) *Observations and Essays on the Statistics of Insanity.* By JOHN THURNAM, M.D., Resident Medical Superintendent of the Retreat, near York. 8vo. pp. 308, with two Appendices, and Tables.

AMONGST the improvements which characterize the modern method of studying medicine, the great attention now paid to pathology is one of the most useful and important. The cultivation of animal chemistry is also another improved feature in the education of medical practitioners. To these may justly be added medical statistics, or the science of numbers as applied to elucidate questions connected with the prevalence, the treatment, and termination of particular diseases. Until very recently, however, statistical investigations were seldom called in to aid the physician in his inquiries; but at present, nothing is more common, especially when opinions differ, than an appeal to the results obtained from numbers, whereby deductions, otherwise speculative, are now reduced almost to a certainty. Entertaining these views, and knowing the practical utility of a numerous array of facts, when well authenticated, and derived from various sources, we therefore hailed with considerable satisfaction the appearance of Dr. Webster's and Dr. Thurnam's "Observations on the Statistics of Insanity," seeing their researches contained much valuable information, and appeared creditable to the authors, on account of the great industry they have shown in collecting materials and carefully examining evidence.

Unless statistical tables are strictly accurate, they are not only valueless, but absolutely injurious to the progress of knowledge, and lead to erroneous conclusions, which deceive the practitioner, and mystify, instead of elucidating, questions under discussion. That improper practices have sometimes prevailed, and statements been made, respecting the mortality and treatment of the insane in certain establishments, which were incorrect, Dr. Thurnam gives the following remarkable example:

"That reports of the results of treatment have been, and still may be, *purposely falsified*, is sufficiently proved by the history of the York Lunatic Asylum, previous to its reform in 1814. It was at that time ascertained, from a minute examination of the records of that institution, that whilst 221 deaths only were officially reported, 365 had actually occurred during the thirty-six years which the asylum had been established, at the commencement of the inquiry into its management in 1813. The deaths of 144 patients had been suppressed in the published reports, and by this means the apparent mortality, for the thirty-seven years 1777—1814, was reduced from 11 per cent., its actual rate, to 7·03 per cent.; or to a lower rate than that which has existed since the reform of the institution, (1814—1840.) Supposing, as there is every reason to believe was the case, that these concealed deaths were added to the recoveries, the effect must have been that of supporting the credit of the institution in a double manner. In proof of the annual reports having been designedly falsified, it is here perhaps only proper to state, that one set of books was kept by the apothecary, and another by the steward, both of which purported to be a correct account of the admissions of the patients, and of how they were disposed of; each of these being in the form of a debtor and creditor account; and thus, if the account was false in one place, it must have been false in more places than one, or else the sums total could not have agreed. The steward's record of the deaths seems to have been tolerably accurate; but, in the face of this, erroneous statements were, year after year, published in the York newspapers, which were only detected upon being compared with the parochial register of the burials from the asylum, in the churchyard of St. Olave's."

We trust and believe that similar proceedings do not now prevail in any asylum; nevertheless, we cordially agree with Dr. Thurnam in the opinion expressed, that until a sufficient examining and superintending body is organized, to which annual reports may be regularly submitted, we can only rely, as the author confidently hopes we are justified in relying at the present day, upon the good faith and integrity of the officers and governors of public institutions.

Besides accuracy, it is also of the utmost importance that the method of calculating the proportion of recoveries and mortality should be simple and of easy attainment. Besides, if one uniform system of registration was adopted in all establishments for the reception and treatment of the insane, the cumulative value of such tables would be thus greatly increased, whilst the deductions, being derived from a much larger series of data, their practical application and utility would be thereby considerably augmented.

Having made these preliminary observations, we proceed, in the first place, to examine the observations of Dr. Webster on the "Statistics and Pathology of Mental Diseases." We would premise that Dr. Webster's opportunities for investigation have been great. His connexion, as governor, with Bethlehem Hospital, has given him free access to the archives of that institution, and he has, with a praiseworthy industry, examined these documents, and has given the profession the result of his investigations, in the papers before us. We consider the *data* which Dr. Webster has given us highly valuable; and it is only by a careful induction from such facts that we shall be enabled satisfactorily to trace the relationship between certain disordered conditions of the mind, and these charges on the organic structure of the nervous system, which experienced men maintain are the invariable cause of deranged mind.

In the 26th and 28th volumes of the "Transactions of the Royal Medical and Chirurgical Society of London," Dr. Webster published two papers on the "Statistics of Bethlehem Hospital," and the "Pathology of Mental Diseases," which contained several points of interest; and as some notice of the questions therein discussed may be acceptable to our readers, we subjoin a short abstract of the facts stated by the author, and the conclusions he has deduced in the two papers read to the society. Before, however, doing so, it ought to be kept in remembrance, that the author's observations apply especially to Bethlehem Hospital, as the archives of that establishment, and the dissections there made, were the sources from whence he makes the statements promulgated.

According to the ancient records, fortunately still preserved in Bethlem Hospital, it appears the total number of lunatic patients admitted, discharged cured, or died, during five different periods of twenty years each, ending the 31st of December, were respectively—

| In 20 Years, ending | Number Admitted. | Number Cured. | Number Died. |
|---|---|---|---|
| 1762 | 3286 | 1069 or $32\frac{1}{4}$ per cent. | 714 or $21\frac{2}{3}$ per cent. |
| 1782 | 3945 | 1366 or $34\frac{1}{2}$ per cent. | 560 or $13\frac{1}{5}$ per cent. |
| 1802 | 3906 | 1379 or $35\frac{1}{3}$ per cent. | 203 or $5\frac{1}{5}$ per cent. |
| 1822 | 2149 | 892 or $41\frac{1}{2}$ per cent. | 111 or $5\frac{1}{6}$ per cent. |
| 1852 | 4484 | 2269 or $51\frac{1}{2}$ per cent. | 224 or $5\frac{1}{14}$ per cent. |
| Totals . | 17,690 | 6975 or $39\frac{1}{3}$ per cent. | 1812 or $10\frac{1}{4}$ per cent. |

By the above statement, the number of insane patients received into Bethlehem Hospital during the latter half of the eighteenth century appears to have varied very little from the previous average, although, during the early part of the present, the actual numbers admitted indicate a material diminution; but at the time when this considerable defalcation in the admissions was noticed at Bethlehem Hospital, so great an increase, compared with the amount of insane patients previously received into the wards of St. Luke's, then took place, that the augmented number at the latter, almost counterbalance the decrease reported at the former institution.

According to an authentic statement in the author's possession, the total number of lunatic patients received into St. Luke's Hospital for the twenty years ending the 31st December, 1802, was 3987, whilst the admissions increased to 5346 during the twenty years ending the 31st December, 1822; although from that date to the 31st December, 1842, they again fell to 4044. From these conclusive facts, it cannot be asserted, that mental alienation prevailed less frequently in London, during the early part of the present century, than it did previously, or has done more recently; notwithstanding fewer insane patients were admitted into the most extensive of the only two public lunatic asylums situated in the metropolis.

The uniformly increasing proportion of patients discharged cured from Bethlehem Hospital, as well as the diminished ratio of mortality in that establishment, are points which well deserve attention; more particularly, as it appears the annual amount of cures have not only steadily advanced, but the comparative number of deaths have also remarkably diminished, since the middle of the last century. For instance, the ratio of recoveries during the first twenty years embraced in the preceding statement, was only $32\frac{1}{4}$ per cent. on the admissions, whilst it rose to $51\frac{1}{2}$ in every hundred patients admitted during a similar number of years ending the 31st December, 1842; at the same time that the amount of deaths actually decreased from $21\frac{2}{3}$ per cent. to $5\frac{1}{14}$ per cent., or less than one-fourth the previous average, after an interval of nearly a century. The difference will, however, appear even more marked, when the results met with during three years in the middle of the last century are compared with similar results reported to have occurred in the three years which terminated on the 31st December, 1842; they were as follows:—

| | Admitted. | Cured. | Died. |
|---|---|---|---|
| In 1750-51-52 | 462 . . . | 145 or $31\frac{1}{2}$ per cent. . . . | 118 or $25\frac{1}{2}$ per cent. |
| In 1840-41-42 | 897 . . . | 492 or near 55 per cent. . . | 51 or $5\frac{5}{8}$ per cent. |

Connected with this important question, it appears the rate of mortality among the patients treated at Old Bethlehem Hospital was so considerable during the year 1753 as to exceed the total number of recoveries, the former being fifty, whilst only forty-three patients were discharged cured; and, as the admissions in that year amounted to 150, the recoveries were at the rate of $28\frac{2}{3}$ per cent., whilst the deaths rose so high as $33\frac{1}{3}$ per cent., being exactly one-third the number of admissions. This very unusual mortality was, however, not altogether owing to insanity, since small-pox then prevailed among the patients, as it likewise did in 1770, when the number of deaths and recoveries were equal, each being 60 to 213 admissions; or 28 per cent. in both cases.

Mental diseases are much more prevalent in this country among women than men. With the view of showing that this opinion is correct, the author gives the subjoined table of curable patients admitted into Bethlehem Hospital during the twenty years ending the 31st December, 1842:—

| Admitted. | | Cured. | | Died. | |
|---|---|---|---|---|---|
| M. | F. | M. | F. | M. | F. |
| 1782 | 2622 . . . . . | 823 | 1446 . . . . . | 112 | 112 |
| or | | or | or | or | or |
| 47 per cent. more females than males. | | $46\frac{1}{5}$ per cent. | $55\frac{1}{7}$ per cent. | $6\frac{1}{4}$ per cent. | $4\frac{1}{4}$ per cent. |

Reasoning from these facts, no doubt can exist regarding the greater frequency of mental alienation among females than males; indeed, the excess of insane women admitted at Bethlehem Hospital is shown to have been 47 per cent., and as the same facilities regarding the admission of patients into that institution prevail, without any reference to sex, provided the cases are recent, the above results must be considered conclusive. A similar opinion is likewise fully borne out by the number of insane patients of each sex admitted into St. Luke's Hospital, during the same period of twenty years, to which reference has just been made; since 1734, lunatic male patients were received into the wards of that charity, from the 31st December, 1822, to the 31st December, 1842; whilst the number of insane females then admitted amounted to 2310, or $33\frac{1}{5}$ per cent. more of the latter than the former sex.

But this greater liability of females than males to diseases of the mind, is at the same time accompanied by other peculiarities worthy of notice—viz., that mania is not only a more common complaint, but more curable, and proves less fatal to women than to men. This conclusion is confirmed by tables which indicate, that the ratio of recoveries from attacks of insanity was nine per cent. in female more than in male patients; whilst in the latter sex, the number of deaths exceeded by two per cent. the rate of mortality met with in the former.

The propensity to commit suicide appears so prevalent a feature in cases of mania, that the attendants upon insane patients cannot be too careful with those showing any symptoms of the kind, lest such a calamitous event should supervene. However, it is satisfactory to know, that instances of self-destruction are now much less frequently met with than formerly, notwithstanding the fact, that patients enjoy, at present, greater freedom, are more frequently engaged in varied occupations, and even sometimes are allowed to use dangerous tools in their respective handicrafts, than in the olden time, when restraint and coercion were more commonly employed. In those days of darkness, when confinement in cells, chains, and personal punishments, were used even as ordinary means of management in cases of lunacy, suicides were more frequent in Bethlehem Hospital than at the present day, when a more humane, and much better mode of treatment is happily pursued.

In proof of this opinion, the author states that eighteen cases of self-destruction occurred in old Bethlehem, from the 1st of January, 1750, to the first of January, 1770, six being male patients, and twelve female; and as the total number of lunatics admitted into that hospital, during the above period, amounted to 3629 patients, there consequently occurred one suicide in about every 202 admissions. Now, however, when restraint, instead of being the rule, as formerly, constitutes the exception, it is gratifying to know that the proportion of suicides has very much diminished. For example, during the twenty years ending the 31st of December, 1842, notwithstanding the total admissions, including 150 incurable, and 122 criminal lunatics, amounted to 4676, only five suicides occurred in this royal hospital, being one case in every 925 insane patients admitted, or less than one-fourth the average number met with among the lunatics confined in old Bethlehem, during the middle of the eighteenth century. It is also worthy of notice, that all the five examples alluded to were women, which singular circumstance, coupled with the previous remarks respecting the suicides reported in the middle of the last century, clearly indicates that a greater propensity to the crime of self-murder prevails in female than in male lunatics.

Another peculiarity may be also mentioned, to show that restraint and strict confinement of lunatic patients do not always insure additional security to the victims of insanity, but even the contrary. According to the records of the charity, more lunatics formerly escaped from old Bethlehem than in the present day, when a strait-waistcoat is almost unknown, and the treatment adopted is quite different. At this institution, during the twenty years ending the 1st of January, 1770, to use an expression met with in the official documents, forty-four male and eleven female lunatics actually "ran away" from the hospital; being one escape in every sixty-six patients admitted. Compared with this authentic statement, it is satisfactory to find, that the number of lunatics reported to have escaped during the twenty years ending the 31st December, 1842, were eleven men and five women, being only one escape in every 292 admissions, or less than one-fourth the previous amount.

From the preceding statement, it appears that a stronger disposition to escape from the confinement to which lunatics were formerly subjected in this asylum, prevailed among the male than the female patients, although the latter class were the most numerous; whilst the propensity to commit suicide, according to the same evidence, was just the reverse. These conclusions are interesting; and seeing they are not peculiar to any particular period, but the uniform results met with in Bethlehem Hospital, during a long series of years, and are deduced from a large number of insane persons of both sexes placed under similar circumstances, they deserve attention from the philanthropist and physician, when directing the treatment of patients afflicted with that greatest of all human calamities, mental alienation.

Another point of interest in this inquiry is the influence which particular seasons of the year exert upon mental affections, as illustrated by the author in the following table of. admissions, &c., at Bethlehem Hospital during twenty-two years:

| Season of the year. | Admitted. | | | Discharged cured. | | | Died. | | |
|---|---|---|---|---|---|---|---|---|---|
| | Males. | Females. | Total. | Males. | Females. | Total. | Males. | Females. | Total. |
| 1st Quar.—Jan. Feb. March | 451 | 649 | 1,100 | 139 | 257 | 396 | 39 | 35 | 74 |
| 2nd Quar.—April, May, June | 545 | 842 | 1,387 | 215 | 375 | 590 | 21 | 34 | 55 |
| 3rd Quar.—July, Aug. Sept. | 551 | 798 | 1,349 | 265 | 410 | 675 | 32 | 30 | 62 |
| 4th Quar.—Oct. Nov. Dec. | 471 | 668 | 1,139 | 333 | 561 | 894 | 32 | 32 | 64 |
| Totals. | 2018 | 2955 | 4974 | 952 | 1603 | 2555 | 124 | 131 | 255 |

According to the above statement, a much larger number of insane patients were received during the second and third quarters than at any other period of the year—that is, when the temperature of the weather increased, so did mental diseases become more frequent. For example, during the second and third quarters of the period referred to, the total curable lunatics admitted into Bethlehem Hospital amounted to 2736; whereas, during the first and fourth quarters, or in the cold season, the actual number was only 2293; being a dimunition of 497 patients, or 22 per cent. less upon the whole admissions. In regard to particular months, it should be noticed that the greatest number of curable lunatics admitted, took place during May—the fewest in January.

Respecting the curability of insanity, it also appears that fewer insane patients were discharged cured during the early part of the year, than in the autumnal months, or towards the approach of winter. For instance, in the first and second quarters, the number of cures were under the average proportion, only 986 lunatics having been discharged convalescent, during that period; whereas, during the last two quarters, 1569 patients left the institution free from mental disease, thus making 583 more recoveries, or an increase of 57 per cent. on the total number of cures reported during the latter, than in the first six months of the twenty-two years comprehended in the previous table.

With reference to the mortality of mental diseases, the same document shows that not only the relative proportion of deaths to the total admissions was larger during the first than in any subsequent quarter, but the actual number of cases terminating fatally during the former period, exceeded the ratio of ony other three months of the same series, whilst the fewest deaths occurred during the months of April, May, and June; when, as already stated, more insane patients were admitted into Bethlehem Hospital than at any other season.

Supported by these data, the physician may rationally conclude that as the temperature of the weather diminishes, and the year draws to a close, so may he give a more favourable opinion respecting the progress of cases of insanity. At the same time, seeing mental affections are in a greater degree prevalent during summer than in winter, every exciting cause, whether physical or moral, ought to be then carefully guarded against, particularly in persons who have been previously afflicted with mental alienation.

Another important question is, the pathology of insanity, which has long occupied the attention of physicians; and, however much has been recently effected in this department, especially by the French pathologists, many points connected with the subject yet require further elucidation. With the view of adding to the stock of information already possessed on this interesting point of medical science, the author gives a synopsis of *one hundred and eight* dissections made at Bethlehem Hospital by Mr. Lawrence, the present distinguished surgeon of that institution, which, not only on that account possess more than an ordinary value, but are otherwise deserving of publication. As our limits, however, do not permit giving any of the autopsies in detail, we must refer those of our readers who are desirous of further information on the subject, to the papers published in the Medico-Chirurgical Transactions; we therefore can only now give a short abridgment of the pathological changes met with in the brain and membranes of the one hundred and eight autopsies which Dr. Webster brought under the notice of the society. Of these, infiltration of the pia mater was observed in ninety-two cases. Turgidity of the blood-vessels existed in eighty-nine. Fluid was effused in the ventricles in sixty-seven. Effusion had taken place at the base of the brain in thirty-nine. There was thickening and opacity of the arachnoid coat in thirty-two. Bloody points were observed on the cut surfaces of the medullary substance in twenty-seven. The colour of the brain appeared changed in nineteen; and in seventeen cases, blood was effused within the cranium. These data indicate unequivocally, that the morbid alterations of structure, characteristic of insanity, which pathologists may expect to find in a majority of cases, will be, infiltration of the pia mater, turgidity of the blood-vessels, and effusion of fluid in the ventricles.

Besides these diseased appearances, various other alterations of structure were met with in particular patients; such as effusion of pus on the brain; changed consistence of its texture; greater dryness than usual of the membranes; flattening, a shrunk, or a swollen state of the organ itself; with other changes different from a normal condition.

Although diseased alterations of structure were not so frequently met with in the organs of the chest, as in the brain and its membranes, nevertheless, eighty-five insane persons in the one hundred and eight dissections described, exhibited changes of a morbid description in the thorax. Indeed, the apparent cause of death, in many of the patients, could be clearly traced to disease in the organs of respiration. Of the eighty-five instances of pectoral disease met with, on examining the bodies after death, fifty-nine cases showed either recent or old adhesions in the chest, and thirty-eight had the lungs consolidated. In thirty-six, suppuration had commenced. In nineteen, the pleura, or lungs, bore marks of recent or previous inflammation. In twenty cases, there was effusion of lymph into the pleura, &c. In nine, considerable effusion into the bronchi and air passages. In nine, the lining membrane of the trachea and bronchi was deep red. In fourteen, tubercles were met with. In eleven, the lungs had assumed a dark or blackish tint. And in nine, the lungs did not collapse when the chest was opened. Besides these diseased alterations of structure, others were noticed, which it is unnecessary at present to particularize.

Respecting the morbid appearances which the abdominal viscera exhibited on dissection, the instances of diseased alteration of structure in any of the patients were so few, as scarcely to admit of many remarks. However, it is right to state, that the liver was found to be affected in ten cases; dropsical effusion had taken place in five patients; and in seven cases, there appeared decided marks of recent and violent inflammation of the contents of the abdomen; in two of which examples, the intestines had actually given way, so as to allow fæcal matter to escape into the peritoneal cavity.

In conclusion, the author alludes cursorily to the important question which now occupies many pathologists, regarding the rationale of the diseased appearances usually met with in the brains of lunatics on dissection after death; namely, whether the morbid alterations of structure, then observed, be the cause, or only the consequence of the patient's previous mental malady. In short, whether the opinions promulgated by the section of pathological physicians, denominated "the Anatomists," or the views entertained by the other party, "the Vitalists," be the true doctrine. The former considering that the diseased alterations of structure observed in the brain produce the attacks of insanity, whilst the latter confidently assert the contrary. Respecting the decision of such important questions, Dr. Webster, although he leaves to others more competent than himself to give an opinion, confesses the numerous illustrations he has detailed, the facts recorded in medical works, as well as the reasoning of authors upon insanity, greatly preponderate in favour of the Anatomists, whose conclusions, in his judgment, at least, appear to be the most rational, and perfectly consistent with the present advanced state of pathological knowledge respecting mental diseases.

We now proceed to Dr. Thurnam's work. In the first section of his observations, the author discusses at some length the application of the terms usually employed in statistical investigations, the accuracy of the reported results, as well as the methods of calculating the proportion of recoveries, and the mean annual mortality in respect of the average population, or mean numbers resident in an institution; and, lastly, the periods over which the observations of reporters should extend. On each of these heads, the reader may consult with advantage the work of the author; but as our space for extracts is limited, we proceed to consider the circumstances influencing the results of treatment, and the methods of exhibiting the proportions of recoveries and mortality.

Regarding the sex of insane patients, the author says—

"That the probability of recovery is greater in women than in men, though the reverse of the opinion entertained by Dr. Burrows, may now be regarded as established; and it is fully supported by a recent inquiry of my own, into the statistics of different asylums of this and other countries. With two exceptions, hereafter to be adverted to, in every institution the statistics of which I have examined, in which the experience has extended over more than a very short period of years, the proportion of recoveries in women has exceeded, often to a great extent, that in men. Thus, in the asylum at Glasgow, taking the entire period of its operation, the recoveries in women, as the table (in a previous page) will show, have exceeded those in men by 4 per cent.; at Belfast by 5, at Lancaster by 7, at Armagh by 10, at Woodbridge by 12, at Worcester, U.S., by 19, at Siegburg by 19, at the Bethlem Hospital, amongst the selected " curable" patients, by 20, at the Retreat, York, by 20, at Schleswig by 22, at Charenton by 23, and at the York Lunatic Asylum by 28 per cent."—Pp. 27, 28.

The only two asylums in which, during any considerable period, Dr. Thurnam has found the recoveries amongst men to have actually exceeded those amongst women, is Hanwell, and the Bloomingdale Asylum, New York. In the former establishment, the recoveries in favour of the male sex were five per cent., whereas, in the American institution, taking a period of more than twenty years, the recoveries amongst men actually exceeded those amongst women by twenty-eight per cent.

Again, with regard to the proportion of deaths amongst the insane, the author remarks :—

" A still greater difference in the rate of mortality of the two sexes is nearly always to be noted. As is well known, there is an excess in the general mortality of this country on the side of males; but this does not exceed 5 or 6 per cent. The relative difference is enormously greater in the insane, in nearly every institution the statistics of which I have had an opportunity of examining on this point. Thus, at the York Lunatic Asylum, and at St. Luke's Hospital, the mortality amongst men has been nearly double that amongst women; there having been an excess on the side of the men of 93 per cent. in the former, and of 96 per cent. in the latter institution. As may be ascertained from the table (in a previous page), the excess of mortality on the side of males amounted to 72 per cent. at Hanwell; to 71 per cent. amongst the " curable" patients at Bethlem; to 63 per cent. in the Metropolitan Licensed Asylums for paupers, and to 57 per cent. in those for private patients; to 57 per cent. at Glasgow; to 56 per cent. at Lancaster; to 46 per cent. at Woodbridge; to 34 per cent. at the Retreat, York; to 29 per cent. at Siegburg, near Bonn; to 13 per cent. at Worcester, U.S.; and to 9 per cent. at Schleswig, Holstein.

" The only British institution I am acquainted with, in which the mortality of the women has exceeded that of the men, is the asylum at Belfast, in which it appears, from Dt. Stewart's reports, that in the fifteen years from the opening of the establishment to the present time, 1844, the mortality of females has exceeded that of males by 12 per cent. For the twelve years, however, ending 1841, the mortality was at precisely the same rate in the two sexes; and it is hardly to be doubted but that a longer experience will yet exhibit a preponderance of deaths on the side of the men."—Pp. 30, 31.

As it will materially assist practitioners in forming their prognosis respecting patients afflicted with mental disease, we think that all statistical tables contained in reports emanating from hospitals for the insane, should contain triple columns, headed " Males," " Females," and " Total." If this system were universally adopted, much valuable information would be procured; and having an extensive and uniformly arranged series of data, the physician would then be able to speak more confidently than hitherto respecting some questions still in dispute.

Seeing the age of a patient exerts a very decided influence both upon the proportion of the recoveries and the mortality of the insane, all statistical tables should contain minute information upon this essential point; and knowing that the probability of recovery is greatest in the young, whilst it undergoes a very regular diminution as age advances, the tables should be arranged according to quinquennial periods of life, up to the age of twenty; but after that period, the arrangement according to decennial periods may be adopted, as sufficiently minute. In illustration of the utility of such an arrangement, the author gives a table of the proportion of recoveries per cent. of the admissions at different ages at the Retreat, the York Asylum, and Bethlehem Hospital. Subsequently another table, indicating the mean annual mortality of patients resident at different ages is supplied; but we would particularly direct attention to the form adopted at the Retreat, which the author strongly recommends, and advises that it should enter into the annual report of every hospital for the insane.

Of all the circumstances which affect the comparison of the recoveries and mortality of lunatics, the stage of duration of the disease is, practically speaking, the most important; for it has long been well known, that there is a greater probability of recovery in recent cases, than in those of protracted duration; whilst the longer the mental malady continues, so will the probability of ultimate recovery constantly diminish. Indeed, after the lapse of twelve months, although recoveries may and do still occur, every physician well knows that the probability of such an event, in the great majority of cases, rapidly decreases. The number of attacks exerts likewise considerable influence; besides, the duration of treatment, as also the methods of cure, whether medical or moral, which are pursued in different lunatic establishments, influence results. In constructing statistical tables, these points should therefore be kept in remembrance, as they furnish most useful information, from whence deductions may be drawn of great practical importance.

The Association of Medical Officers of Hospitals for the Insane, has done much towards obtaining a well-digested method of registration, and in the collection of results of various kinds, classified in a numerical form. In pursuance of this object, the association some time ago distributed a form of register which they wished to be adopted by those of their members who were connected with asylums. If the form which was ultimately agreed upon be fully carried out, by even a limited number of medical officers, much good may be expected ultimately to result from its introduction; and we confidently anticipate that such an aggregate mass of information will be thereby procured, as to prove of great practical utility to the profession. The necessity for some uniform method of obtaining as full histories as may be possible of all cases admitted into lunatic asylums is most evident, as some of the tables still in use in several establishments are exceedingly brief, not to say defective. Being advocates for the diffusion of correct and uniform statistical information on so important a subject as insanity, we coincide in opinion with the author, that it is very desirable the forms employed should undergo a general revision, and be made to embrace all the facts required by a complete and detailed register, which should be introduced into every public hospital for the insane throughout the British empire.

In the second chapter of his work, Dr. Thurnam makes some judicious remarks respecting the healthiness of the locality in which an asylum should be situated, the influence of climate, soil, as well as on the means of exercising, occupying, and amusing the inmates. Unquestionably, all lunatic asylums should be placed upon elevated ground, and should likewise command cheerful prospects. The soil should be dry, and there should be always a most ample supply of water. These circumstances contribute materially towards the salubrity of any public institution, and of course must influence the data contained in returns emanating from such establishments. In regard to the external character of hospitals and asylums of large size, Dr. Thurnam thinks the H form to be the best adapted, in order to secure the complete separation of the sexes, proper classification, and ready inspection by the superintending officers. But for smaller establishments, intended to contain from 80 to 150 patients, the plan which may be called the K form appears to be the most appropriate; whilst it possesses the merit, not to be undervalued, of presenting a more ornamental and cheerful-looking structure.

The ventilation, lighting, warmth, and cleanliness of the apartments occupied by insane patients, exercise likewise considerable influence, as also the number of lunatics which should be treated in one asylum. The best authorities agree that a preference should be given to establishments of moderate size; because in hospitals for such large numbers as 500, 700, or even 1,000 insane persons, the maintenance of effective ventilation and proper cleanliness must be far more difficult. Although many concurrent causes may, in some institutions, operate upon the health of the inmates, physicians are not justified in attributiug the higher mortality of some asylums solely to their greater average population; still Dr. Thurnam thinks—

"It is still highly probable that it is in part owing to this circumstance that the mean mortality of those county lunatic asylums which have the largest population, as Wakefield with a population of 400, Lancaster with one of 600, and Hanwell with one of nearly 1000, is much above the average of asylums of the same class, which have been similar periods in operation, but which receive a smaller number of patients."—P. 87.

Nevertheless, however correct this opinion may appear, it is equally consistent with experience, that the crowded state of a moderate-sized establishment produces an even more unfavourable effect on the health of its inmates, than does the congregation of a very large number of persons in a building which would otherwise seem sufficiently extensive.

Plentiful and nutritious food, in the opinion of all persons conversant with the management of lunatics, is a point of essential importance in every asylum. To show the advantages of properly feeding insane patients, Dr. Thurnam classifies seven asylums into two separate groups; in one of which the diet was, at the time to which the table refers, considerably above, and in the other, considerably below, the average diet of the country asylums, taken as a class. The first group includes the three asylums for the counties of Nottingham, Stafford, and Gloucester; the second, those of Lancaster, the west riding of York, Suffolk, and Middlesex. The author then remarks:—

"The difference in the amount of the diet in the two groups, is, as will be seen, very considerable. Exclusive of vegetables, the solid food, consisting of meat and cheese, and of puddings, bread and other farinaceous articles, amounted, on an average, to 225 ounces in the first, and to only 150½ ounces per week in the second group. In the first group, as regards solid food, the diet was 50 per cent. better than that in the second. The difference in the relative amount of solid animal food, considered separately, was still greater, and amounted to 130 per cent.: the weekly allowance of meat and cheese being, on an average, 46 ounces in the first, and only 19½ ounces in the second group. Of course the quantity of soup, porridge, milk, and other fluids was greater in the four asylums in which the solid food was at the minimum, than in the other three; these amounting, on an average, to 15½ pints in the one, and to not more than 10 pints per week in the other, group. Still this greater amount of fluids could by no means compensate for so great a difference in the quantity of solid food, and especially in that of meat. In the first group, also, the quantity of beer allowed was much greater than in the second; the quantity being two pints daily in the one, and, for the most part, not more than half a pint in the other.

"That in institutions in every way of the same character, there should be so large a difference in the quantity and description of the food, is of itself sufficiently surprising: and would, without any reference to results, appear to call for inquiry and equalization, upon some ascertained principles, as regards the requirements of the insane in this respect. But should it be found, as from the table (in a previous page) appears highly probable, that the diet of the insane does in truth exert a material influence upon the results of treatment,—upon the proportion of the recoveries and the mortality,—the necessity for some such inquiry into, and equalization of, the diet in different asylums and hospitals for the insane becomes still more obvious. In the three asylums with the more liberal diet, we find that the recoveries averaged 43.7 per cent., and that the mean mortality was 9.35 per cent.; whilst in the four institutions in which the diet was less liberal and nutritious, the recoveries only averaged 36.75 per cent., and the mean mortality was as high as 14.54 per cent. It must not, however, be forgotten that there may be, and no doubt are, other circumstances in the condition of these asylums, which materially influence the results of treatment, and which will thus explain many of the discrepancies in the results which the table exhibits; but, though this is the case, I cannot but conclude that the amount of difference which does exist, is in great measure dependent upon the difference in the diet."—Pp. 95—97.

The treatment of the insane, whether medical or moral, exerts also a very decided influence on the progress and ultimate result in many cases of insanity; seeing that, in well-conducted asylums, where a judicious plan of medical treatment is pursued, a larger proportion of recoveries appears to be reported than in other institutions. It is also generally found, that a good system of moral management is accompanied by corresponding excellencies, and a bad system, by corresponding defects; particularly in the degree of attention which is paid to exercise, employment, and to the personal comfort and cleanliness of the patients. In illustration of such principles, Dr. Thurnam thus refers to the history of the York Lunatic Asylum, which portrays the internal condition of that establishment at different periods:—

| "York Lunatic Asylum. | Mean annual Mortality per cent. Resident. |
|---|---|
| "*Before Reform of* 1814—37 years, 1777—1814 | 11. |
| First Physician: 31 years, 1777—1809 | 9.5 |
| Second Physician: 6 years, 1809—1814 | 14.8 |
| *Since Reform of* 1814.—29.66 years, 1814—1844 | 7.24 |

"More than a quarter of a century has now elapsed since the reform alluded to took place; and during this period, (29.66 years, 1814—44,) which is one sufficient to ensure accuracy, the mean annual mortality has not exceeded 7.24 per cent.; a rate which it has observed, with remarkably slight deviations, through the entire period. This is a mortality very much higher than that observed at the Retreat, where it has only amounted (1796—1843) to 4.7 per cent.; but which, judging from the experience of institutions similarly circumstanced, must still be regarded as a favourable one for insane persons under confinement. That the mortality, under the superintendence of the two first physicians, during the extended period of 37 years, amounting as it did to 11. per cent., should have exceeded that which has since been observed at the rate of 40. per cent., must, I think, without doubt, be at least principally attributed to the difference which then existed in the management of the institution, and in the treatment of the patients."—P. 113.

Other questions respecting the statistics of insanity are also investigated in the volume now under review, but our limited space precludes us from enlarging further upon this part of the author's publication, which contains, as already acknowledged, much useful information upon a subject hitherto but partially studied by the profession. Nevertheless, giving every credit to the author's industry in collecting his materials, we think they might have been arranged in a more simple and intelligible form, so as to be better adapted to the generality of medical readers. We would also remark, notwithstanding the various tables comprised in Dr. Thurnam's really valuable publication, no statistical statement is given illustrative of the influence of particular seasons of the year upon mental affections; that is, did the temperature of the weather increase or diminish such disorders, and whether insanity was more curable, or proved more fatal, during one period or month of the year than at another? These are questions of some practical importance, and, we think, if tables containing such data were properly constructed and analyzed, the medical practitioner would be occasionally assisted in forming his prognosis: at all events, he would thus get additional and useful information. We throw out the hint for the consideration of the superintendents of lunatic asylums; and Dr. Thurnam must pardon us when we say, that we wish his work had contained tables of the above description.

Besides the subject more especially investigated by Dr. Thurnam, the volume now before us contains three essays on the liability to insanity. One, in regard to the relative liability of the two sexes to that disease; another, respecting the different ages of insane patients; and the third discusses the tendency to insanity in the Society of Friends. We cannot enter upon the examination of these different essays, however much they might repay perusal, but must confine the few remarks we can now make to the first of these productions; the more so, as the subject it embraces is one which has recently attracted considerable attention, both in this country as also on the continent, and especially as some difference of opinion still prevails respecting the question at issue. Many physicians—at least, in modern times—have maintained that insanity is more prevalent amongst women than amongst men. Burrows, Esquirol, Prichard, Copland, Brown, and Millingen, and, indeed, nearly every recent writer on insanity, have supported the former doctrine, whilst the author of the present essay advocates a contrary opinion, which also prevailed even so early as the times of Aretæus and Cælius Aurelianus. Dr. Thurnam, in support of his views, that more men become insane than women, makes some ingenious remarks respecting the general population of the country; and he endeavours to show, besides other reasons, that the opinion of women being more liable to insanity than men, originated in an erroneous method of statistical analysis. He also thinks the method which Esquirol—a great authority on all questions connected with mental disease—adopted of investigating this subject was fallacious, particularly in his having compared with each other the *existing*, instead of the *occurring*, cases of insanity in the two sexes. Nevertheless, Dr. Thurnam, who appears only anxious to ascertain the truth respecting the point now in dispute, observes—

"The only two institutions, however, that I am acquainted with in this country, in which there has been any material excess of females admitted during extended periods, are the hospitals of Bethlem and St. Luke; and in these there has been, during different and extended periods, an excess of women admitted amounting to 20, 30, and even 45 per cent. This, however, may depend on local circumstances peculiar to the metropolis; and does not, consequently, in any degree establish Dr. Haslam's opinion, that 'in our own climate, women are more frequently afflicted with insanity than men;' a statement which has been recently repeated by Dr. Webster in his remarks 'On the Statistics of Bethlem Hospital.'"—P. 146.

Notwithstanding the apparent force of the arguments Dr. Thurnam brings forward in support of the position he has taken up, we confess our opinion coincides with that of Esquirol and the other authorities already mentioned. In illustration of these views, reference may be made to a recent Report of the Metropolitan Commissioners in Lunacy, wherein it is stated, that in asylums of all descriptions in England and Wales—but not including Bethlehem Hospital, as that institution is independent of the commissioners' jurisdiction—there were under treatment on the 1st of January, 1844, 5751 female, and only 5521 male lunatics. It should also be taken into account, in order to arrive at correct opinions in such an inquiry, that as regards the higher and middle ranks of society in this country, it is well known many women belonging to such classes, instead of being sent so frequently as men into public and private asylums, are, from various causes, more likely to be detained at home during their mental affliction, where they sooner become entirely dependent than the other sex, and consequently are oftener treated with greater indulgence by friends and relatives. However, until additional statistical information is supplied to decide this point, it may be considered to remain, in some degree, *sub judice*, notwithstanding the reasoning and numerous facts the author has adduced. Nevertheless, we repeat the opinion we have previously expressed, in order to draw the attention of medical statisticians more especially to the subject; at the same time, we think it is important to remark, that the Report of the Commissioners in Lunacy, presented to parliament during last session, like the former similar document already quoted, supplies farther evidence adverse to Dr. Thurnam's particular views. In this official paper, the commissioners report that the number of insane persons confined in asylums, hospitals, and licensed houses, within their jurisdiction, amounted to 7009 female, and only 6217 male lunatics, pauper as well as private patients being included; thus making an excess of 12.73 per cent. of insane women more than men at that period resident in the public establishments throughout England and Wales.

Appended to Dr. Thurnam's publication, is an elaborate report respecting the history and statistics of the Retreat near York, of which institution the author is the zealous medical superintendent. This document deserves perusal, and shows how much the Society of Friends have done towards alleviating one of the greatest afflictions incident to humanity. But our critical remarks must now draw to a close. However, before taking leave of Dr. Thurnam, we would again observe, although we do not wholly coincide in several of the conclusions at which he has arrived, and think the great mass of valuable materials the author has collected might have been otherwise arranged, no one can doubt the writer's merit and industry, and that he deserves the thanks of the profession for this useful contribution to medical science.

